# Multidisciplinary Management and Autologous Skin Grafting in a Patient with Severe Burns: A Case Study

**DOI:** 10.3390/medicina60081201

**Published:** 2024-07-24

**Authors:** Giovanni Cangelosi, Stefano Mancin, Diego Bei, Eleonora Clementi, Paola Pantanetti, Gabriele Caggianelli, Fabio Petrelli

**Affiliations:** 1 Units of Diabetology, ASUR Marche, 63900 Fermo, Italy; dr.paolapantanetti@gmail.com; 2 Humanitas Research Hospital, 20089 Rozzano, Italy; stefano.mancin@humanitas.it; 3 School of Nursing, Polytechnic University of Marche, 60121 Ancona, Italy; diegobei@hotmail.it (D.B.); ele21072000@gmail.com (E.C.); 4 Azienda Ospedaliera San Giovanni Addolorata, 00184 Rome, Italy; caggianelligabriele@gmail.com; 5 School of Medicinal and Health Products Sciences, University of Camerino, 62032 Camerino, Italy; fabio.petrelli@unicam.it

**Keywords:** burn, skin grafting, multidisciplinary team, wound care, case study

## Abstract

*Background*: Heat burns are a prevalent type of trauma. Rapid and effective treatment is crucial for deep burns to minimize complications. Autologous skin grafting is a highly effective treatment for full-thickness burns. A multidisciplinary team plays a vital role in managing burn patients undergoing skin grafting, from initial contact to outpatient follow-up. *Case Summary*: This case study involves a 56-year-old patient who suffered burns on 60% of his body following an alcohol explosion on an open flame. The patient underwent autologous skin grafting at a Major Burn Center. Initial symptoms included severe pain and immobility, but the patient remained alert and breathed spontaneously. The diagnosis was a loss of epidermis and dermis with burns covering 60–69% of the total body surface area (TBSA) and third-degree burns covering 10% TBSA. Post-discharge, the patient showed significant improvement, with complete healing of the grafts and partial resolution of other lesions. Six months after the intervention, the patient significantly improved his autonomy and mobility. *Conclusions*: This case highlights the importance of burn prevention and the critical role of multidisciplinary teams in the entire care pathway of burn patients. Appropriate diagnosis, complete treatment, and continuous multidisciplinary support are essential to prevent complications and ensure recovery.

## 1. Introduction

Burns remain a significant global health concern, representing the fourth leading cause of injury worldwide, with nearly 11 million incidents annually, resulting in approximately 180,000 deaths due to complications [1,2,3,4]. The morbidity and mortality associated with burns are particularly high in low- and middle-income countries, where access to specialized burn care is limited. Burns are traditionally classified into four degrees (I, II, III, and IV) based on the extent of tissue damage. This classification ranges from superficial epidermal injuries (first degree) to full-thickness injuries involving deeper anatomical structures such as muscles and bones (fourth degree) [5,6,7]. However, contemporary clinical practice more commonly categorizes burns into superficial (involving only the epidermis) and deep (extending into the dermis or beyond) based on their severity and required treatment.

The primary etiological factors for burns include thermal agents (such as flames, hot liquids, and hot surfaces), electrical sources (like direct contact with electrical currents), and chemical agents (including strong acids or alkalis) [6,7,8,9,10,11,12,13,14]. These etiological differences necessitate varied approaches to initial treatment and long-term care.

The preferred treatment approach for burns is largely conservative, focusing on wound care, infection prevention, and pain management [15,16,17,18]. However, for deeper burns, particularly from grade IIB onwards, skin grafting becomes a critical intervention. Skin grafting techniques include autologous (using the patient’s own skin), allogeneic (using donor skin), and xenoplastic (using skin from other species) grafts [19]. Among these, autologous grafting is highly effective due to its lower rejection rates and better integration with the host tissue, significantly improving healing outcomes [20,21,22,23,24,25,26].

In this clinical context, the multidisciplinary team plays a pivotal and strategic role in the successful implementation of individualized care plans across all phases of burn treatment. Their responsibilities extend from acute emergency care, where they are often the first point of contact, through to long-term rehabilitation and follow-up. This team, composed of doctors, nurses, physiotherapists, psychologists, and other healthcare professionals, ensures comprehensive care, including wound management, pain control, psychological support, and patient education. This holistic approach is crucial for optimizing recovery and improving the quality of life for burn patients. The coordination and collaboration among multidisciplinary team members are essential for addressing the complex needs of burn patients, demonstrating the value of integrated and personalized care strategies [27,28,29,30,31,32].

This case study aims to provide a detailed description of the comprehensive management and recovery of a patient who suffered severe burns following an alcohol explosion over an open flame and to explore the patient’s experience. This case study also includes a qualitative section enriched with a semi-structured interview aimed at capturing the patient’s perspective and treatment outcomes, highlighting the crucial role of multidisciplinary care in the overall recovery process.

## 2. Materials and Methods

This study was conducted by applying the Case Study Observational Research framework of Morgan et al. [33], a structured approach designed to ensure a comprehensive and systematic analysis of phenomena within their real-life contexts. The framework begins with the clear definition of research questions and objectives, followed by the purposive selection of the case to ensure relevance and depth of analysis. Data collection is conducted using multiple methods, including interviews, observations, and document analysis, enabling triangulation to enhance the validity and reliability of the results. Data analysis is performed through an iterative process of thematic analysis. Furthermore, the framework emphasizes reflexivity and ethical considerations throughout the research process, ensuring both transparency and ethical integrity.

### 2.1. Setting 

This study was conducted at a tertiary hospital in Italy, specialized in the treatment of severe burns. The hospital facility features an advanced burn center and a highly qualified multidisciplinary team, including burn specialists such as plastic surgeons, intensive care physicians, specialized nurses, and other healthcare professionals. The hospital is equipped with advanced technologies for continuous patient monitoring and is capable of performing surgical interventions for debridement and skin grafting, as well as providing the intensive care necessary for the treatment of extensive and complex burns. 

### 2.2. Data Collection

In order to fully capture all aspects of the patient care experience, data collection was conducted using both observational and non-observational methods.

With regard to observational data, this research was conducted by an experienced member of the healthcare team who conducted non-participatory research. Researchers used detailed notes to document the care processes.

With regard to the non-observational data, a research member of the multidisciplinary team conducted semi-structured interviews with the patient from the preliminary observations. These interviews aimed to explore and validate the observed themes, providing insights into the patient’s experiences and the effectiveness of the care provided (Supplementary File S1). To complement the data obtained from the observations and interviews, an analysis of relevant literature and patient records was conducted.

### 2.3. Ethical Approval

This study was conducted following national and international regulations and the Declaration of Helsinki. The subject of this case study was informed about this study’s objective; oral and recorded (the patient could not write for reasons due to the burn) consent was preliminary obtained in compliance with all privacy regulations (Art. 13 EU Regulation 679/2016) before administering the survey and taking a single photo. According to Italian national and regional regulations, for the present model study, the approval from the relevant ethics committee was tacit consent after a specific statement was provided (n. 2220/2223). The data were processed anonymously.

## 3. Results

### 3.1. Case History

The case involves a 56-year-old Italian man who was generally in good health before the incident. His medical history includes arterial hypertension, managed pharmacologically only during the winter season for approximately five years, and a right saphenectomy performed two years prior to the incident. The patient was allergic to amoxicillin and nimesulide.

The incident occurred while the patient was using alcohol to ignite a grill over an open flame in his garden, resulting in an explosion. This incident caused third-degree burns, classified according to the American Burn Association (ABA) burn severity scale, over a total body surface area (TBSA) of 60–69%. The event took place in the presence of his family, and his daughter, a nurse, was the first to intervene. She likely minimized the damage by cooling the wound with cold water and promptly calling for emergency assistance. Following the incident, the main reported symptoms were intense pain, burning sensation, immobility, and inability to manage self-care. The patient underwent a clinical procedure involving autologous skin grafting, and the clinical course was marked by significant pain, assessed using the Numerical Rating Scale (NRS) with a score of 7. Pain management involved the use of a morphine syringe pump for approximately seven days. Pain during dressings, particularly on the upper and lower limbs, was managed through sedation following an anesthesiologic consultation.

### 3.2. Therapeutic Interventions and Diagnostic Evaluations

The patient was admitted to the Major Burn Center of our hospital on 8 August 2023 and placed under reserved prognosis due to the extensive nature of the burns. During the hospitalization, multidisciplinary therapeutic interventions were carried out.

Pharmacological therapy primarily included the continuous infusion of albumin and rehydrating fluids until 20 August 2023 and the use of protein supplements to support anabolic function. The patient was continuously monitored during the first week, with spontaneous respiration assisted by oxygen therapy at 2 L per minute. The fluid balance was often negative in the initial weeks. Multidisciplinary consultations were requested with nephrologists, physiatrists, infectious disease specialists, and anesthetists for pain management, initially treated with a morphine infusion pump for the first seven days.

During wound dressings, local anesthesia was administered at the specific request of the patient, following an anesthesiologic consultation. On 29 August 2023 and 8 September 2023, the patient underwent two surgical procedures for autologous skin grafts, which were both successful from both reconstructive and general clinical perspectives. Specific antibiotic prophylaxis was prescribed according to reference guidelines to prevent infections. The central venous catheter (CVC) was removed on 19 September 2023.

Multidisciplinary care interventions focused on promoting the patient’s autonomy, ensuring adequate hydration and nutrition, managing dressings, recording vital signs (VS), providing bedside hygiene, and planning overall multidisciplinary care according to the reference organizational model for optimal functional and emotional recovery. The patient was encouraged daily to ambulate and perform common daily activities, such as washing, eating, drinking, and moving independently in bed, following a gradual schedule. Vital signs were generally stable, except for an episode of hypotension after the second surgical procedure. The multidisciplinary team emphasized fluid and protein intake, especially in the final part of the hospitalization and after the CVC removal. Urine output and bowel function were monitored daily and stimulated as necessary.

The main diagnostic and instrumental examinations performed during the hospitalization included a chest X-ray and an electrocardiogram (ECG) upon admission. Culture tests from skin swabs taken on 21 August 2023 revealed positive results for *Acinetobacter Baumannii* and *Candida Parapsilosis*. Blood cultures, performed for a body temperature (BT) > 38.5 °C on 21 August 2023, with two samples from peripheral veins and one from the central vein, were all negative and were repeated on 4 September 2023 for hyperpyrexia, again being negative. The patient was also subjected to additional culture tests, including a urine culture on 8 August 2023 (negative), a rectal swab on 6 September 2023 (negative), and a nasal swab for COVID-19 on 9 August 2023 (negative).

**Figure 1:**
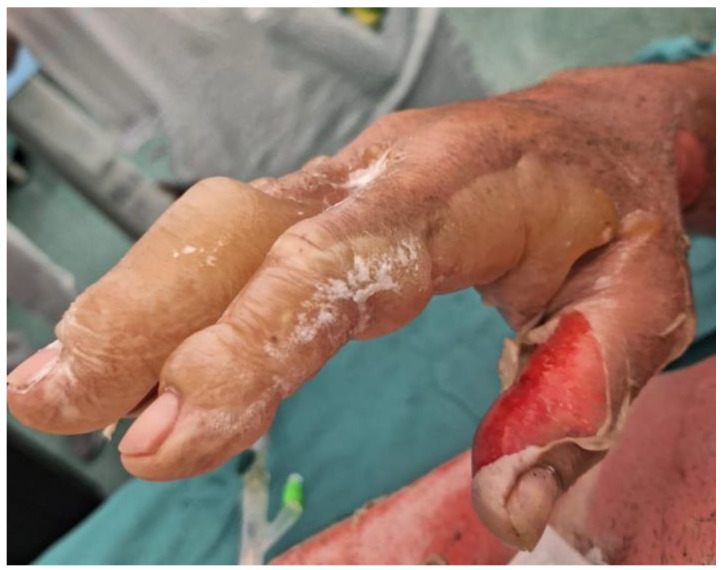
Burns immediately after the incident (right hand).

**Figure 2:**
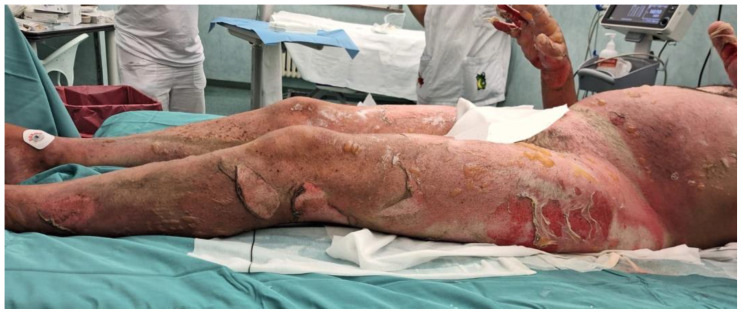
Burns immediately after the incident (left leg).

Figure 1 and Figure 2 show the burns immediately after the incident (15 August 2023) and the subsequent healing of the skin areas where the grafts were performed. The depth and severity of the injuries are visible, particularly on the lower left limb, where the epidermal layer is entirely detached from the dermal layer. There are areas with a reddish coloration, indicating severe and deep tissue damage. The perilesional skin is visibly red and warm, with a moderate number of blisters on the lower abdomen and left thigh.

### 3.3. Hospital Discharge 

The patient was discharged from our Major Burn Center on 24 September 2023 with stable vital signs, partial autonomy in ambulation, and the ability to perform activities of daily living. Pain was well controlled, with a NRS score 2, and was managed with a prescription of 1 g of paracetamol as needed (up to three times a day). A course of wound care dressings was prescribed to be performed at the wound care nursing clinic. 

**Figure 3:**
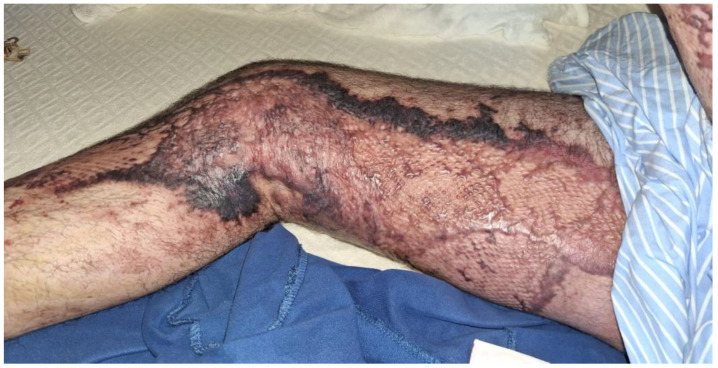
Skin condition at discharge.

Figure 3 illustrates the clinical condition of the skin at discharge. After two months of treatment, the skin graft on the right leg was nearly healed, distinguishable from the surrounding damaged skin by its distinct mottled texture.

### 3.4. Post-Discharge Treatment and Follow-Up

The patient underwent a regimen of bi-weekly dressings for the first month post-discharge, followed by weekly dressings. The dressing procedures involved cleansing with a solution of 0.057 g sodium hypochlorite (active chlorine 0.055 g), followed by the application of fusidic acid/sodium fusidate 20 mg/g and betamethasone (betamethasone dipropionate) 0.05% ointments on the lesions. Secondary dressings with sterile gauzes and non-compressive bandages were then applied.

For areas with dry or dehydrated skin, a moisturizing cream was applied twice daily, and the patient was advised to wear only cotton clothing. Throughout the follow-up period, the patient underwent weekly rehabilitative physiotherapy sessions. By December 2023, significant improvement was noted, with the majority of the skin lesions being completely healed and without pain. The patient continued with weekly dressings using adhesive polyurethane foam dressings, facilitating complete healing within a month and significantly improving joint mobility compared to the initial phase of outpatient follow-up.

By February 2024, the grafts were visibly healed and no longer required further intervention, except for the use of moisturizing and soothing creams, which the patient managed independently.

### 3.5. Qualitative Interview

The results of this case study show the complexity and intensity of multidisciplinary care to manage severe burns. The patient care pathway was characterized by several key issues: the critical role of immediate first aid, the continuous and adaptive nature of multidisciplinary interventions, and the long-term challenges of rehabilitation and recovery.

#### 3.5.1. Critical Role of Immediate First Aid

The first care given to the patient was critical in mitigating the severity of the burns. The patient recounted the incident as follows:

*“It happened while I was preparing the barbecue for a grill. I wet the charcoal with alcohol and put the bottle under my left arm without putting it aside because I was sure the flames wouldn’t reach the bottle, but instead, within a few seconds, it caught fire and exploded. At first, I thought the damage was only to my legs, and I thought I could extinguish the fire myself, but it wasn’t the case. I was wearing a T-shirt and shorts that were melting, so I took off everything and remained practically naked. Then, my wife and daughter, who is studying to be a nurse, arrived, and they put me under the cold shower. At the MBC, they told me that cold water prevented the burn from going deep.”*

#### 3.5.2. Continuous and Adaptive Multidisciplinary Interventions

During the hospital stay, the patient underwent intensive multidisciplinary care. The patient described it as follows:

*“I was hospitalized for 38 days. I was always lying on the bed because my legs, arms, pelvis, and trunk were covered in bandages. I couldn’t do anything; I could only endure the pain. Every 2/3 days, they changed my dressings, but I couldn’t move. I wasn’t independent at all. At first, I couldn’t even dress myself, but now I can. I am about 80/90% independent; I can move my arms fairly well. I could do something, but I had pain and discomfort. It’s essential to have someone to help you.”*

The patient received continuous monitoring and care by a team of specialists, including nephrologists, physiatrists, infectious disease specialists, and anesthetists, to manage pain and other complications. Surgical procedures for autologous skin grafts were successfully performed, supported by a detailed post-operative care plan.

#### 3.5.3. The Long-Term Challenges of Rehabilitation and Recovery

Rehabilitation continued for a long period after discharge from hospital. The patient shared the following:

*“After the accident, I haven’t returned to work yet; I‘m still on sick leave. I certainly think about the problems with my hands. I still have some difficulty with my left hand. I may need to wear gloves, protective devices at work. The MBC assured me that I would return to my previous life in about two years. I hope to return to what I used to do. The issue is that until I reach a point where I can manage my work, I can’t quantify the time. Being on sick leave for six months, I‘ve explained the situation. For some time now, the skin on my hands is still thin, and since my legs were also burned, we’ll have to assess if I‘ll be able to stand for the entire duration required by the company during working hours.”*

The patient also faced problems in regaining physical strength and mobility:

*“I used to ride motorcycles in my free time… I haven’t fully regained the lost muscle tone during the long hospitalization. Also, the sensitivity in my hands isn’t like before… the physiotherapists at the MBC gave me instructions on what I needed to do, and I followed their instructions at home. Unfortunately, I expected a faster recovery, but I perceive it as very slow. I improved the functionality of my arm; before, I couldn’t move it, but now it’s no longer a problem, and it doesn’t hurt, although it’s still full of scars that cause pain when touched.”*

The patient’s recovery journey was also characterized by emotional and psychological challenges:

*“Physically, I feel reasonably well, mentally sometimes not… due to the wounds I managed to touch my legs only after five months… The healing process continues, but it’s slow; compared to the beginning, my legs are doing better.”*

## 4. Discussion

Burns represent a significant public health issue in both low- and middle-income countries and high-income countries, with substantial economic and social consequences for the individuals and communities involved. Burns entail considerable healthcare costs, work absences, and long-term disabilities, having an impact at both personal and community levels [34,35,36]. In developed countries, the reduction in burn incidence has been associated with the implementation of targeted prevention plans, which have emphasized the importance of structured and evidence-based preventive interventions [4]. These interventions include public awareness campaigns, improvements in domestic and workplace safety regulations, and the adoption of advanced protective technologies [35,36].

In this study, despite the clinical complexities associated with burns, the patient showed significant improvements thanks to the support of a multidisciplinary team composed of doctors, nurses, physiotherapists, and other healthcare professionals. This highlights the importance of an integrated approach to burn management, covering all stages from acute care to rehabilitation [37]. The use of advanced healthcare technologies, such as autologous skin grafts, facilitated faster and more complete healing, reducing the risks of complications associated with allogenic or xenoplastic grafts [38]. This outcome was supported by a detailed post-discharge dressing protocol, including specific solutions for cleaning, the application of antimicrobial agents, and wound protection, which were crucial for preventing infections and promoting optimal healing [39]. The multidisciplinary approach also included a continuum of care aimed at functional recovery and autonomy in daily life activities. This is fundamental in anticipation of the post-discharge phase and the patient’s return home [40]. The development of structured programs promoting continuity of care between hospital and community is of paramount importance to improve the management of outpatient and community care [41]. These programs should be designed to address the complexities of burns and improve clinical outcomes through personalized and coordinated multidisciplinary care [42]. 

The results of the semi-structured interview highlight the complexity of healthcare for patients with severe burns. Immediate first aid intervention, such as the application of cold water, was essential to limit the damage caused by the severe burns. The analysis of the issues also revealed that the constant support of the multidisciplinary team was decisive for the management of the patient’s pain, as well as for the successful outcome of skin grafting procedures or the resolution of complications. Finally, the recovery aspect of the patient required intensive rehabilitation, and the patient had to cope with physical and psychological difficulties. These data highlight the need for a holistic and well-coordinated approach integrating comprehensive multidisciplinary treatment and long-term rehabilitation support.

This study also underscores the urgency of further research and developments in integrated burn management. It is essential to continuously improve clinical practices and optimize outcomes for affected patients through therapeutic innovations and evidence-based treatment strategies. Future research should focus on the effectiveness of multidisciplinary approaches and the integration of new technologies for burn treatment, with the goal of improving patients’ quality of life and reducing the incidence of long-term complications.

### Limitations

This case study, while providing valuable insights into the multidisciplinary management of burn injuries, is inherently limited by its nature. Being focused on a single patient, the findings may not be universally applicable due to individual variability in response to treatment. Moreover, the detailed documentation of all procedures and interventions is challenging in a case study format. This includes the comprehensive description of clinical protocols and the integration of visual aids, such as images, which are crucial for demonstrating clinical progress and outcomes. Despite these limitations, this study highlights important aspects of integrated care that can inform future studies and clinical practices.

## 5. Conclusions

Burn injuries remain a significant public health issue worldwide. This case study highlights the importance of a multidisciplinary approach in managing burn patients, demonstrating significant improvements in patient outcomes through coordinated care, involving doctors, nurses, physiotherapists, and other healthcare professionals. The findings underscore the necessity of structured prevention plans and the continuity of care programs that bridge hospital and community services to enhance the overall management of burn injuries. Although this study is based on a single case, it provides valuable insights that can inform and improve clinical practices in burn care.

## Data Availability

Data supporting this research are available upon request from the corresponding author.

## Supplementary Materials

The following supporting information can be downloaded at: https://www.mdpi.com/article/10.3390/medicina60081201/s1, File S1: Interview Guide.
