# Supplementary material for: Multidisciplinary Management and Autologous Skin Grafting in a Patient with Severe Burns: A Case Study
Source: Medicina (Kaunas). 2024 Jul 24;60(8):1201. doi: 10.3390/medicina60081201 (PMC11356184; doi:10.3390/medicina60081201)
Supplement: Supplementary file 1 [file medicina-60-01201-s001.zip › medicina-3072529-supplementary.pdf]

SUPPLEMENTARY FILE

Interview Guide

1. How did the accident happen?
2. After the intervention, what was your level of autonomy in the hospital during your stay? And after discharge?
3. Did you work before the accident? After the accident, were you able to continue your work? Were you offered a change in position?
4. Did you have any hobbies/passions before the accident?
5. Can you ride motorcycles more now?
6. Were you trained to do exercises independently at home? Do you have physiotherapy sessions with a professional?
7. Did you take any medication before the intervention? After the intervention, did the doctor prescribe any medication for you?
8. How do you feel today?
